# Personal Safety during the COVID-19 Pandemic: Realities and Perspectives of Healthcare Workers in Latin America †

**DOI:** 10.3390/ijerph17082798

**Published:** 2020-04-18

**Authors:** Diego Delgado, Fernando Wyss Quintana, Gonzalo Perez, Alvaro Sosa Liprandi, Carlos Ponte-Negretti, Ivan Mendoza, Adrian Baranchuk

**Affiliations:** 1 Division of Cardiology, University Health Network, Toronto, ON M5G2N5, Canada; 2 Division of Cardiology, Cardiovascular Services and Technology of Guatemala—Cardiosolutions, Guatemala 01010, Guatemala; fernandowyss@gmail.com; 3 Division of Cardiology, Olivos Clinic, Buenos Aires B16022ABQ, Argentina; gonzaeperez@gmail.com; 4 Division of Cardiology, Sanatorio Güemes, Buenos Aires C1188AAF, Argentina; asosaliprandi@gmail.com; 5 Cardiometabolic Unit, Instituto Médico La Floresta, Caracas 1090, Venezuela; ciponten@gmail.com; 6 Tropical Cardiology Central, University of Venezuela, Caracas 1060, Venezuela; Imivanjm@gmail.com; 7 Division of Cardiology, Kingston Health Science Center, Queen’s University, Kingston, ON K7L2V7, Canada; barancha@kgh.kari.net

**Keywords:** coronavirus, Latin America, healthcare, safety

## Abstract

Healthcare workers exposed to coronavirus (COVID-19) may not have adequate access to personal protective equipment (PPE), safety procedures, and diagnostic protocols. Our objective was to evaluate the reality and perceptions about personal safety among healthcare workers in Latin America. This is a cross-sectional, online survey-based study administered to 936 healthcare professionals in Latin America from 31 March 2020 to 4 April 2020. A 12-item structured questionnaire was developed. A total of 936 healthcare workers completed the online survey. Of them, 899 (95.1%) were physicians, 28 (2.9%) were nurses, and 18 (1.9%) were allied health professionals. Access to protective equipment was as follows: gel hand sanitizer (*n* = 889; 95%), disposable gloves (*n* = 853; 91.1%), disposable gowns (*n* = 630; 67.3%), disposable surgical masks (785; 83.9%), N95 masks (*n* = 516; 56.1%), and facial protective shields (*n* = 305; 32.6%). The vast majority (*n* = 707; 75.5%) had access to personal safety policies and procedures, and 699 (74.7%) participants had access to diagnostic algorithms. On a 1-to-10 Likert scale, the participants expressed limited human resources support (4.92 ± 0.2; mean ± SD), physical integrity protection in the workplace (5.5 ± 0.1; mean ± SD), and support from public health authorities (5.01 ± 0.12; mean ± SD). Healthcare workers in Latin America had limited access to essential PPE and support from healthcare authorities during the COVID-19 pandemic.

## 1. Introduction

The coronavirus (COVID-19) outbreak has fundamentally changed the world and, consequently, is changing the reality of healthcare workers. This pandemic is creating profound changes in governments, the global economy, and healthcare systems.

Based on current evidence, the COVID-19 virus is transmitted between people through close contact and droplets [1]. The people most at risk of infection are those who are in close contact with a COVID-19 patient or who care for COVID-19 patients. Healthcare workers are at significant risk of acquiring the infection; therefore, they are required to protect themselves and prevent transmission in the healthcare setting. 

Precautions to be implemented by healthcare workers caring for patients with COVID-19 include using appropriate personal protective equipment (PPE). The World Health Organization (WHO) and other national and international public health authorities recommend implementing safety protocols for healthcare workers [2]. However, basic protective equipment and safety protocols are not always available in many medical institutions dealing with COVID-19 patients. 

Many medical institutions around the world do not have access to an appropriate number of human resources and diagnostic/therapeutic protocols to care for admitted and ambulatory patients suffering from COVID-19. 

According to the Pan American Health Organization (PAHO) and the WHO, the number of confirmed cases in Latin America is 26,486, and the number of deaths is 858 as of April 4, 2020 [3]. Unfortunately, there is a significant discrepancy in regards to access to PPE, human resources, and healthcare policies in countries in the region of the Americas. The speed with which COVID-19 is spreading across the word calls for an assessment of the reality of healthcare workers exposed to COVID-19 patients.

The purpose of this study was to evaluate the reality and perceptions about personal safety among healthcare workers practicing in countries of Latin America during the current COVID-19 outbreak. 

## 2. Methods

### 2.1. Study Design

This is a cross-sectional, online survey-based study administered to healthcare professionals in Latin America. A 12-item questionnaire was developed and distributed using Google Forms. Participants were recruited through social networking websites and applications (Twitter, Instagram, Facebook, LinkedIn, and WhatsApp) and from an existing database of the Inter-American Society of Cardiology (IASC). The questionnaire was conducted from 31 March 2020 and until 4 April 2020. The survey was delivered in Spanish, as the targeted study participants were in Spanish-speaking countries of Latin America.

Participants were able to complete the survey only once and were allowed to terminate the survey at any time they desired. The survey was anonymous and confidential. An introductory paragraph outlining the purpose of the study was posted along with the survey. The survey was prepared by members of the COVID-19 Working Group of IASC.

### 2.2. Outcomes

A 12-item structured questionnaire was developed to evaluate participants’ reality and perceptions regarding personal safety.

The study questionnaire comprised four sections. Section 1 had five items that collected demographic information of the responders. This included age by segments (18–14, 25–35, 36–45, 46–55, >55 years), sex (male or female), occupation (physician, nurse, other healthcare professional), type of practice (hospital, private, or both), and geographic location. Section 2 comprised four items and was designed to evaluate access to PPE (gel hand sanitizer, disposable gloves, disposable gowns, disposable masks, N95 masks, facial protective shields), access to personal safety policies and procedures (yes or no), access to COVID-19 diagnostic and treatment algorithms (yes or no), access to telemedicine to evaluate and follow up with patients (yes or no), and institutional support with human resources in case healthcare workers are sick (10-point Likert scale; 0 = no resources, 10 = full access to resources). Section 3 comprised two items designed to evaluate participants’ perceptions about their medical institutions taking all necessary measurements to protect physical integrity in the workplace (10-point Likert scale; 0 = no support, 10 = full support) and participants’ perceptions regarding their local public health authorities taking all necessary measurements to protect physical integrity in the workplace (10-point Likert scale; 0 = no support, 10 = full support).

### 2.3. Statistical Analysis

Descriptive statistics, frequencies, and percentages were used to summarize data. 

## 3. Results

### 3.1. Demographic Characteristics

**Figure 1:**
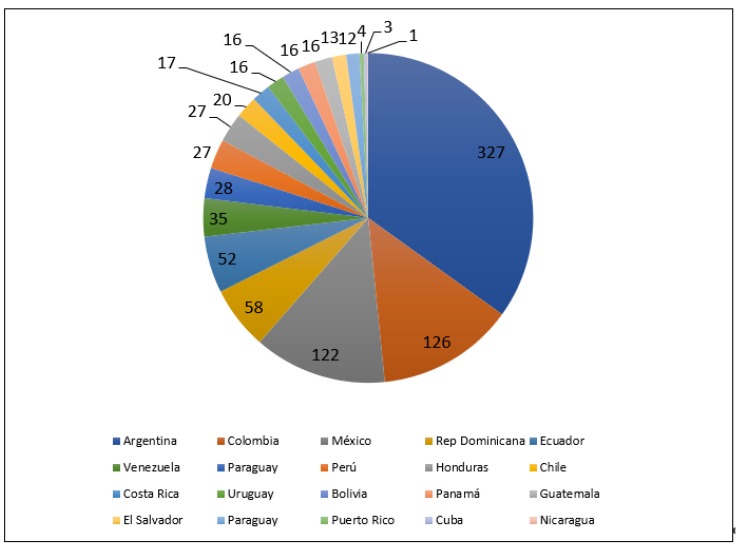
Distribution of participants by geography location.

A total of 936 healthcare workers completed the online survey. Of them, 890 (95.1%) were physicians, 28 (2.9%) were nurses, and 18 (1.9%) were professionals in other healthcare disciplines. The responders’ medical specialties were not reported in this survey. Most participants were men (*n* = 674; 72%), were aged 36–45 (*n* = 281; 30%), and worked in both hospital-based and private practice (*n* = 448; 47.9%) (Table 1). Figure 1 shows the distribution of the participants by geographic location.

### 3.2. Outcomes of Interest

**Figure 2:**
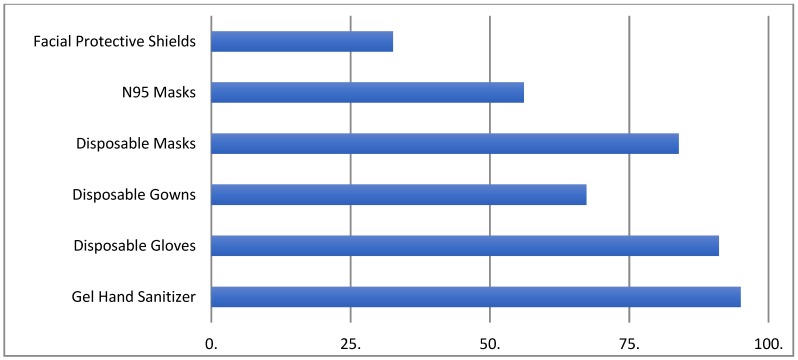
Access to personal protective equipment (PPE). Types of PPEs (%) accessible to healthcare workers.

Participants indicated that they had access to the following essential items: gel hand sanitizer (*n* = 889, 95%), disposable gloves (*n* = 853; 91.1%), disposable gowns (*n* = 630; 67.3%), disposable masks (785 83.9%), N95 masks (*n* = 516; 56.1%), and facial protective shields (*n* = 305; 32.6%) (Figure 2).

In terms of access to personal safety policies and procedures in the workplace, 707 (75.5%) participants responded that they had access, and 229 (24.5%) did not have access. The majority of the participants (699; 74.7%) had access to COVID-19 diagnostic and treatment algorithms, and 237 (25.3%) had no access. Regarding access to telemedicine to evaluate and follow up patients, 572 (61.1%) healthcare workers had access, and 364 (38.9%) did not have access. 

**Figure 3:**
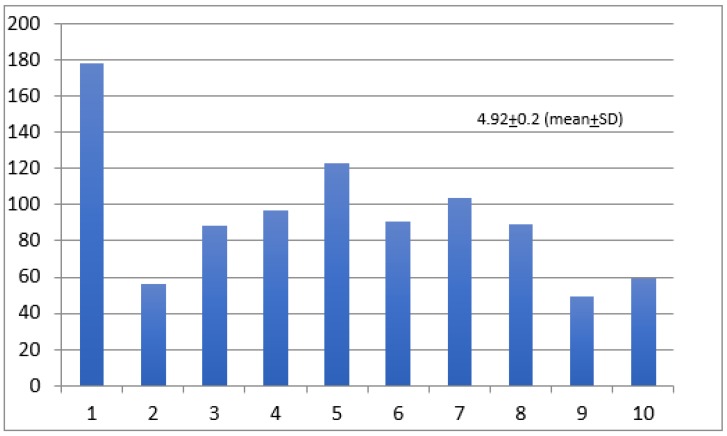
Institutional support with human resources. Likert Scale: 1 = no access to resources, 10 = full access to resources. *Y*-axis = number of responders.

When asked about their own medical institution supporting healthcare workers with additional human resources in case they became sick, the mean ± SD score was 4.92 ± 0.2 on a scale of 1 to 10 (Figure 3).

**Figure 4:**
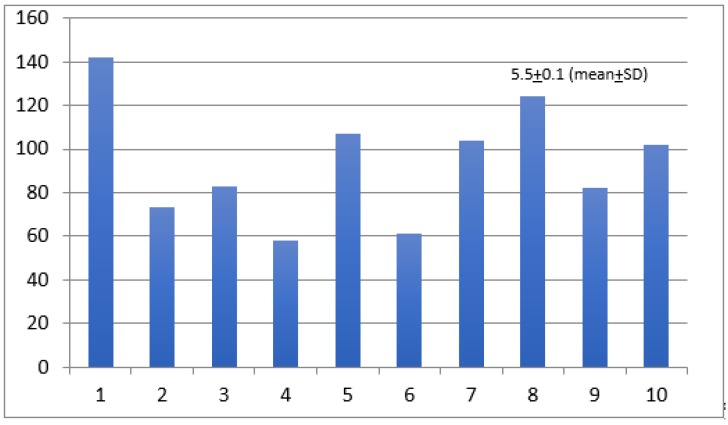
Support from medical institutions in regards to the protection of physical integrity in the workplace. Likert Scale: 1 = no support, 10 = full support. *Y*-axis = number of responders.

**Figure 5:**
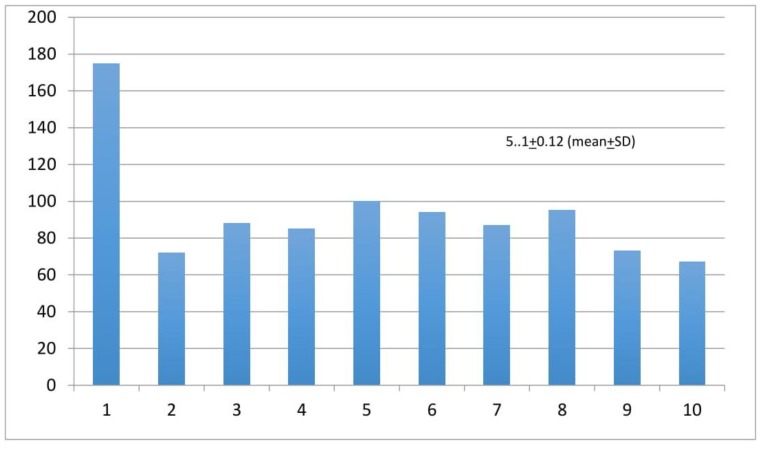
Support from local public health authorities in regards to the protection of physical integrity in the workplace. Likert Scale: 1 = no support, 10 = full support. *Y*-axis = number of responders.

The participants’ perceptions about their medical institutions taking all necessary measurements to protect physical integrity in the workplace was 5.5 ± 0.1 (mean ± SD) (Figure 4). Finally, we asked participants to share their perceptions about their local public health authorities taking all necessary measurements to protect their physical integrity in the workplace. The results show a mean ± SD of 5.01 ± 0.12 (Figure 5).

## 4. Discussion

This cross-sectional online survey enrolled 936 healthcare workers in Latin America. The majority of the responders were physicians actively based in a hospital or private practice in Spanish-speaking countries in North, Central or South America. 

Our study indicates that most of the participants had access to basic PPE; however, there were many healthcare professionals who did not have the required equipment recommended by the WHO, particularly disposable masks and N95 masks. Surprisingly, only 32.6% of the participants had access to facial protective shields. These findings highlight the need for essential PPE to care for suspected and/or confirmed cases of COVID-19.

The WHO, PAHO, and other national and international public health authorities recommend implementing social distancing and self-isolation to mitigate the impact of this disease. Thus, many suspected/confirmed COVID-19 patients and even non-COVID-19 patients do not have access to appropriate medical care during this pandemic. Remote medical monitoring via phone or the internet is an available tool that assists healthcare providers in delivering care to patients at home [4]. However, this technology was not widely available (61.1%) for the healthcare professionals participating in this study.

Exceptional efforts have been made by healthcare workers in Latin America to apply the latest and most effective safety measurements to protect their health in the workplace. However, based on our findings, many of our colleagues do not have safety policies and procedures in place at the workplace.

The perception of healthcare workers about the limited support from medical institutions and local public health authorities in regards to their own safety shows that there is much work to be done in that respect. 

This study’s findings on the reality and perceptions about the safety and resources available for healthcare workers during the COVID-19 pandemic could inform medical institutional authorities about the need for urgent implementation of safety policies and deployment of human resources. The findings of this study could also be used to set priorities in terms of safety and human resources allocation by public health authorities. 

Two cross-sectional surveys on COVID-19 were recently published. One publication addressed the important factors associated with mental health in healthcare workers exposed to COVID-19 [5]. Another study analyzed the general public knowledge and perceptions about the COVID-19 outbreak [6]. To the best of our knowledge, our study is the first cross-sectional survey conducted among healthcare workers in Latin America regarding the reality and perceptions about safety procedures in the workplace. It is important to note that some countries were non- or under-represented in this survey. 

Although knowledge of the disease and updates on COVID-19 among healthcare workers are being given full consideration, the framework to safely implement those recommendations is still lacking. 

## 5. Limitations

This study has several limitations. It was limited in scope. Participants were asked to answer very specific questions that might not cover the complex situation of the personal safety of healthcare professionals. 

No power calculations were undertaken prior to the initiation of the study. However, the purpose of this study was only descriptive and not hypothesis testing. 

Recruitment of participants was based on their willingness to participate and access to social networking websites and applications; therefore, the study population does not encompass participants without those resources. Many countries in Latin America were not represented or poorly represented in the survey. It is possible that most of the responders work in the cardiovascular field where the exposure to critical COVID-19 patients may be limited; therefore, their reality and perspective about COVID-19 could differ from those of other specialists. The inability to determine the universe under this study makes the generalizability of our findings quite limited. 

## 6. Conclusions

Protecting healthcare workers is a public health priority. In this survey study of healthcare professionals working in Latin America, we reported limited access to essential personal protective equipment during the COVID-19 pandemic. The poor perception of healthcare professionals about not having enough support from medical institutions and public health authorities raises the need to urgently implement strategies to protect healthcare workers in the time of the COVID-19 pandemic. 

## Figures and Tables

**Table 1:** Demographics and characteristic of the healthcare workers.

Characteristics	Category	*n* (%)
Age	18–24	0 (0%)
	25–35	183 (19.6)
	35–45	281 (30)
	45–55	266 (38.4)
	>55	204 (21.8)
Sex	Female	262 (27.9)
	Male	674 (72)
Medical Profession	Physicians	890 (95.1)
	Nurses	28 (2.9)
	Other	18 (1.9)
Medical Practice	Hospital-based	321 (34.3)
	Private practice	167 (17.8)
	Both	448 (47.9)
